# 

**Published:** 1844-07

**Authors:** 


### CONTENTS OF No. XXXV

OF THE

### British and Foreign Medical Review.

JULY, 1844.

---

### Part First.—Analytical and Critical Reviews.

PAGE

Art. I.—Clinique Chirurgicale de l'Hôpital de la Pitié. Par J. Lisfranc. Tome troisième . . . . . . 1

Clinical Surgery of the Hospital of La Pitié. By J. Lisfranc. Third Vol.

Art. II.—Report of the Commissioners appointed to take the Census of Ireland for the year 1841. Presented to both Houses of Parliament by Command of Her Majesty . . . . . . 35

Art. III.—Anatomisch Pathologische Opmerkingen over de Ontsteking van eenige Inwendige Deelen van het Oog, en bijzonder over Choroiditis als Oorzaak van Glaucoma. Door J. L. C. Schroeder van der Kolk, Hoogleeraar in de Geneeskunde te Utrecht . . . . . . 48
Anatomico-pathological Remarks on the Inflammation of some internal parts of the Eye, and especially on Choroiditis, as the cause of Glaucoma. By J. L. C. Schroeder van der Kolk, Professor of Medicine at Utrecht.

Art. IV.—On the Nature and Treatment of Tic Douloureux, Sciatica, and other Neuralgic Disorders. By H. Hunt, m.d. . . . . . 56

Art. V.—The Physiology of Inflammation and the Healing Process. By Benjamin Travers, f.r.s. . . . . . . 69

Art. VI. — Recherches, Anatomiques, Physiologiques et Pathologiques sur les Cavités closes, naturelles ou accidentelles, de l'Economie Animale. Par A. Velpeau, &c. . . . . . . 79
Researches, Anatomical, Physiological, and Pathological, on the Close Cavities of the Animal Economy, natural or accidental. By A. Velpeau, &c.

Art. VII.—1. Principles of Medicine: comprising General Pathology and Therapeutics, and a brief general view of Etiology, Nosology, Semeiology, and Prognosis. By Charles J. B. Williams, m.d. f.r.s., Professor of the Principles and Practice of Medicine, and of Clinical Medicine; and First Physician to the Hospital, University College, London, &c. . . 91

2. The actual Process of Nutrition in the Living Structure demonstrated by the Microscope; and the Renewal of the Tissues and Secretions, with the Phenomena and Products of Inflammation, illustrated and established. (Second Series of Experimental Researches.) By William Addison, f.l.s., &c. . ib.

Art. VIII. — On Regimen and Longevity; comprising Materia Alimentaria, National Dietetic Usages, and the Influence of Civilization on Health and the Duration of Life. By John Bell, m.d. . . . . 115

4295

PAGE

ART. IX.—1. On Feigned and Factitious Diseases, chiefly of Soldiers and Seamen; on the means used to simulate or produce them; and on the best modes of discovering Impostors. By HECTOR GAVIN, M.D. . . . 117

2. On the Enlisting, Discharging, and Pensioning of Soldiers, with the official documents on these branches of military duty. By HENRY MARSHALL, F.R.S.E., Deputy-Inspector-General of Army Hospitals . . . ib.

3. Memorial de l'Expert dans la Visite sanitaire des Hommes de Guerre, ou examen des principales questions relatives aux maladies et infirmités qui peuvent donner lieu à l'exemption et à la réforme du service de l'armée de terre, et à leur simulation, provocation, et dissimulation. Par L. FALLOT, D.M., Médecin Principal de l'Armée, etc. . . . . . ib.

ART. X.—1. Anatomico-Chirurgical Observations on Dislocations of the Astragalus. By THOMAS TURNER, Esq. M.R.C.S.L. . . . 124

2. Mémoire sur l'Extirpation de l'Astragale, lu à l'Académie des Sciences dans la Séance du 13 Février, 1843. Par MM. ROGNETTA et FOURNIER-DESCHAMPS, D.M. . . . . . . . ib.

A Memoir on Extirpation of the Astragalus; read before the Academy of Sciences, 13th Feb. 1843. By MM. ROGNETTA and FOURNIER-DESCHAMPS.

ART. XI.—1. Untersuchungen über den Bau des Nerven-systems. Von Dr. B. STILLING und Dr. J. WALLACH. Zweites Heft, enthaltend Untersuchungen über die Textur und Function der Medulla Oblongata. Von Dr. B. STILLING. 134

Researches into the Structure of the Nervous System. By Dr. B. STILLING and Dr. J. WALLACH. The Second Part, containing Researches into the Structure and Function of the Medulla Oblongata.

2. Mikroskopiske Undersögelser af Nervensystemet. Ved ADOLPH. HANNOVER, Lic. Med. . . . . . . . . ib.

Microscopical Investigations of the Nervous System. By Dr. ADOLPH. HANNOVER.

3. Untersuchungen über die Physiologie der Nervenfaser. Von Dr. G. H. MEYER, Privat-docenten zu Tübingen, &c. . . . ib.

Researches into the Physiology of Nervous Fibrils. By Dr. G. H. MEYER, &c.

ART. XII.—Darstellung der Verfassung und Einrichtung der Baumwoll-Spinnerei-Fabriken in Nieder-Oesterreich. Mit besonderer Beziehung auf die moralisch-intellectuelle und physiche Erziehung der daselbst verwendeten Kinder und die diessfalls bestehenden gesetzlichen Vorschriften. Von Dr. JOS. JOH. KNOLZ . . . . . . . . . 147

An Account of the Constitution and Management of the Cotton-mills in Lower Austria. With especial reference to the moral, intellectual, and physical education of the children employed in them, and to the legal regulations subsisting in this respect. By J. J. KNOLZ, M.D.

ART. XIII.—Mémoires de l'Académie Royale de Médecine. Tome Dixième . 152

Memoirs of the Royal Academy of Medicine. Vol. X.

ART. XIV.—1. Contributions to Proleptics. By THOMAS LAYCOCK, M.D., Physician to the York Dispensary, &c. . . . . . . 165

2. Untersuchungen über periodische Vorgänge im gesunden und kranken Organismus des Menschen. Von GEORG SCHWEIG . . . ib.

Inquiries into the Periodic Occurrences in the Healthy and Morbid Organism of Man. By GEORGE SCHWEIG.

3. Instructions pour l'Observation des Phénomènes périodiques. Par M. QUETELET, &c. &c. (Bulletin de l'Académie Royale de Bruxelles, No. i, tom. ix, 1842) ib.

Instructions for the Observations of Periodic Phenomena. By M. QUETELET.

4. Instructions pour l'Observation des Phénomènes périodiques de l'Homme. Par M. SCHWANN. (Bulletin de l'Académie, &c. No. vii, tom. ix) . ib.

Instructions for the Observation of the Periodic Phenomena of Man. By M. SCHWANN. (Bulletins of the Royal Acad. of Brussels, Nos. i and vii, vol. ix.)

PAGE

ART. XV.—1. Natural History, Pathology, and Treatment of the Epidemic Fever at present prevailing in Edinburgh and other Towns. By JOHN ROSE CORMACK, M.D. F.R.S.E., &c. . . . . . 179

2. Remarks on the present Epidemic Fever. By WILLIAM PULTENEY ALISON, M.D. F.R.S.E., &c. . . . . . . ib.

3. Letter on the present Epidemic of Dundee. By J. ARROTT, M.D. . ib.

4. Clinical Observations on Fever. By Professor HENDERSON . . ib.

5. On the Presence of Urea in the Blood in the prevailing Epidemic Fever and in Typhus. By MICHAEL W. TAYLOR, Esq. . . . . ib.

6. Remarks on the Epidemic Fever in Aberdeen during the year 1843. By A. KILGOUR, M.D. . . . . . . ib.

7. On some of the Characters which distinguish the Fever, at present Epidemic, from Typhus Fever. By Prof. HENDERSON . . . . ib.

8. Some Account of the Epidemic Fever prevailing in Glasgow. By DAVID SMITH, M.D. . . . . . . . ib.

9. Notice of a Febrile Disorder which has prevailed at Edinburgh during the summer of 1843. By DAVID CRAIGIE, M.D. . . . . ib.

10. Some Account of the Epidemic Remittent Fever prevalent in Glasgow in 1843. By WILLIAM MACKENZIE, M.D. . . . . . ib.

ART. XVI.—1. The Fourth Annual Report of the Registrar-general of Births, Deaths, and Marriages in England, 1842 . . . . 197

2. The Fifth Annual Report of the Registrar-general of Births, Deaths, and Marriages in England, 1843 . . . . . ib.

3. An Essay on the best modes of representing accurately, by Statistical Returns, the Duration of Life, and the Pressure and Progress of the Causes of Mortality amongst different classes, &c. By EDWIN CHADWICK, Esq. . ib.

ART. XVII.—Précis Analytique sur le Cancer de l'Estomac et sur ses Rapports avec la Gastrite Chronique et les Gastralgies. Par le Docteur BARRAS . 208
Analytical Sketch of Cancer of the Stomach, and of its Relations to Chronic Gastritis and Gastralgia. By Dr. BARRAS.

ART. XVIII.—Outlines of Pathology and Practice of Medicine. By WILLIAM PULTENEY ALISON, M.D. F.R.S.E. . . . . . 213

ART. XIX.—The Influence of Climate and other Agents on the Human Constitution, with reference to the Cause and Prevention of Disease among Seamen; with Observations on Fever in general, and an Account of the Epidemic Fever of Jamaica. By ROBERT ARMSTRONG, M.D. F.R.S. . . 221

ART. XX.—Special Anatomy and Histology. By WILLIAM E. HORNER . 223

ART. XXI.—Two Essays on the Diseases of the Spine: 1, On Angular Curvature of the Spine, and its Treatment; 2, On the Treatment of Lateral Curvature by Gravitation, Lateral Exercise, &c. By R. A. STAFFORD . . 225

ART. XXII.—A Practical Manual, containing a Description of the general Chemical and Microscopical Characters of the Blood, and Secretions of the Human Body, as well as of their Components, including both their Healthy and Diseased States; with the best methods of separating and estimating their Ingredients; also, a succinct account of the various concretions occasionally found in the body, and forming calculi. By JOHN WILLIAM GRIFFITH, M.D., F.L.S., &c. . . . . . . . . . 228

ART. XXIII.—1. Nervous Diseases, arising from Liver and Stomach Complaints, Low Spirits, Indigestion, Gout, and Disorders produced by Tropical Climates; with Cases. By G. R. ROWE, M.D. F.S.A. . . . . 229

2. On some of the most Important Disorders of Women. By G. R. ROWE, M.D. F.S.A. . . . . . . . . . ib.

### PART SECOND.—Bibliographical Notices.

PAGE

ART. I.—Scrofula, its Nature, Causes, and Treatment; and on the Prevention, and Eradication of the Strumous Diathesis. By W. TYLER SMITH, M.B. . 231

ART. II.—An Essay towards a Correct Theory of the Nervous System. By JOHN HARRISON, M.D., &c. . . . . . . 232

ART. III.—Tabulæ ad illustrandam Embryogenesin Hominis et Mammalium, tam naturalem quam abnormem. Auctore W. VROLIK, Med. Doctor, &c. . 233

Plates for the Illustration of the Development, both natural and abnormal of Man and Mammalia. By W. VROLIK, M.D., Professor in the Athenæum at Amsterdam, &c.

ART. IV.—On Superstitions connected with the History and Practice of Medicine and Surgery. By T. J. PETTIGREW, F.R.S. . . . 234

ART. V.—Prize Essay on the Nature and Objects of Medical Science, and the Principles upon which its Study and Practice ought to be founded. By PHILIP HENRY WILLIAMS, M.D. . . . . 235

ART. VI.—An Essay on the Tongue, in Functional Derangement of the Stomach and Bowels. By E. WILLIAMS, M.B. . . . . 236

ART. VII.—The Diseases of Children, their Symptoms and Treatment. A Treatise intended for the use of the Student and Junior Practitioner. By G. A. REES, M.B. . . . . . . . ib.

---

### PART THIRD.—Original Reports and Memoirs.

Report on the Influence of Civilization in lessening the Number and Severity of Diseases. By K. F. H. MARX, M.D., Professor in the University of Göttingen. . . . . . . . 237

Report on the Changes in the Blood in Inflammation, and on the Nature of the Healing Process. By T. WHARTON JONES, F.R.S., Lecturer on Anatomy, Physiology, and Pathology, at the Charing Cross Hospital; Corresponding Member of the Imperial and Royal Society of Physicians and Surgeons of Vienna, &c. . . . . . . . 255
- Changes of the blood in Inflammation . . . . ib.
  - 1. *a*. Changes in the Stagnant Blood . . . . ib.
  - Explanation of the Change in the stagnant Blood . . ib.
  - 2. *a*. Changes in the general Mass of Blood . . . 257
  - 2. *b*. Explanation of the Changes in the general Mass of Blood . 260
- Terminations of Events of Inflammation . . . . 268
  - Nature of Mortification . . . . . ib.
  - Nature of the Healing Process . . . . ib.
  - Microscopically-visible Phenomena attending the Reestablishment of the Circulation . . . . . 269
  - Explanation of the Microscopically-visible Phenomena attending Re-establishment of the Circulation . . . . ib.
  - Exuded Matter . . . . . . 271
  - Absorption of exuded Matter . . . . ib.
  - Development of Organic Elements in the exuded Matter . . ib.
  - Resolution of Inflammation . . . . 279
  - Healing of a Wound . . . . . ib.

OBITUARY—Dr. Barlow . . . . . . 281

BOOKS RECEIVED FOR REVIEW . . . . . 284

